# Analysis of antiquorum-sensing and antibiofilm activity by pomelo peel extract (*Citrus maxima*) on multidrug-resistance *Pseudomonas aeruginosa*

**DOI:** 10.37796/2211-8039.1364

**Published:** 2022-12-01

**Authors:** I Gede Krisna Arim Sadeva, Putri Ayu Wulandari, Aizar Vesa Prasetyo, Luh Putu Nike Wahyuntika, Putu Nindya Krisnadewi Rahadi, I Gede Aswin Parisya Sasmana, I Made Rhamandana Putra, Agus Eka Darwinata

**Affiliations:** a Faculty of Medicine, Udayana University, Denpasar, Bali, Indonesia; b Gadjah Mada University, Indonesia; c Department of Microbiology, Faculty of Medicine, Udayana University, Denpasar, Bali, Indonesia

**Keywords:** Biofilm, *Citrus maxima*, Multidrug resistance, *Pseudomonas aeruginosa*, Quorum sensing

## Abstract

*Pseudomonas aeruginosa* is gram-negative bacteria with high adaptability by forming biofilms and quorum-sensing mechanisms to avoid immune responses and antimicrobial agents which tend to develop into Multidrug Resistance (MDR) related to Healthcare-Associated Infection (HAI) with a prevalence of 3,8% in Indonesia and a mortality of up to 69%. Polyphenol compounds found in pomelo peels (*Citrus maxima*) have been shown to have antibiofilm and antiquorum-sensing effects but are less investigated. Therefore, this study aimed to investigate those effects on MDR *P. aeruginosa*. In vitro study design is performed to evaluate the inhibition effect of ethanolic extract on bacterial growth (Kirby-Bauer test), biofilm formation (biofilm assay), and quorum-sensing activity (pyocyanin and pyoverdine assay) on clinical isolates of MDR and ATCC strain as comparator. Furthermore, we employed computational methods using docking protein analysis. Biofilm formation was significantly inhibited by 71.1% ± 4.4% in MDR (pLasR and *LasI*) by active compounds of *Citrus maxima*. Molecular docking was used to further strengthen this hypothesis, showing no significant differences in bonding energy of polyphenol compounds found in pomelo peel with *LasR* and *LasI* compared to the native ligand and inhibitors. Pomelo peel extract can be considered as a potential therapy for MDR *P. aeruginosa* infection mediated based on its antibiofilm and antiquorum-sensing effects.

## 1. Introduction

Healthcare-Associated Infection (HAI) or Nosocomial Infection (NI) is a group of infectious diseases that are spreading and occur in hospitals or health installations within 48 hours after receiving treatment therapy or 30 days after surgery. Several types of HAI include pneumonia, meningitis, bacteremia, and urinary tract infections that have high morbidity and mortality rates which are increasing every year [1]. The prevalence of HAI cases is up to 7.1% which is equal to four million people in 2016 in European countries [2]. In fact, the prevalence of HAI-related cases in Indonesia reaches 3.8% [3]. Most HAI cases predominantly infected groups of people with immunocompromised conditions, such as ICU patient, which causes the HAI mortality rate to rise to 69% [4].

HAI cases are generally caused by the bacterium *Pseudomonas aeruginosa (P. aeruginosa)* with a prevalence of 29.6% of the total HAI cases in the world in 2017 [5]. *P. aeruginosa* is a gram-negative bacterium as well as an opportunistic pathogen with high adaptability. Several drug therapies, including potent antibiotics, inhibit these pathogens by stopping the growth of bacteria (bacteriostatic) or killing the bacteria (bactericidal). Unfortunately, the misuse of antibacterial agents creates new problems that lead to the formation of bacterial strains that are resistant to these compounds as a result of random mutations in the bacterial DNA genome, known as Multidrug-Resistant Bacteria (MDR) *P. aeruginosa* [6]. A previous study has identified MDR bacteria in dr. Soeradji Tirtonegoro Center Hospital, Central Java, which is dominated by *Pseudomonas* sp. (26.9%) strains [7].

*P. aeruginosa* can resist most classes of antibiotics through the formation of complex organic structures known as biofilms. The protective nature of the biofilm allows *P. aeruginosa* to evade the immune response and create a suitable environment for bacterial growth so that bacteria can survive UV light exposure, changes in pH, changes in temperature, and the effects of disinfectants [8]. This formation of biofilms is influenced by a quorum sensing (QS) mechanism, the ability of bacteria to communicate with other bacteria through signal transduction using a molecule called an autoinducer, such as acyl-homoserine lactone (AHL) [9].

QS mechanism involves two circuits of AHL, RhII-RhIR, and LasI-LasR. LasI-LasR initiates the entire cascade of QS or known as quorum quenching (QQ). These proteins act as transcription factors that activate many genes, including *rhlI* and *rhlR* through catalyzed production of N-3-oxo-dodecanoyl homoserine lactone (3OC12-HSL), the diffusible QS signal [10]. RhIR protein is an autoinducer synthetase receptor that controls virulence factors of *P. aeruginosa* including biofilm development [11]. Most of the antibiotic classes currently used as therapy, such as β-lactams, aminoglycosides, and fluoroquinolones antibiotics, do not target QS activity or biofilms. Therefore, most of the MDR *P. aeruginosa* does not yet have resistance to compounds that target the QS mechanism and biofilms, so this type of therapy has great potential for further development [5].

Various compounds have been known to inhibit one of these mechanisms. However, anti-QS and antibiofilm agents generally require high production costs and use specific materials that are difficult to obtain, such as 2-benzene dicarboxylic acid, which must be produced synthetically [12]. Various natural compounds have also been shown to inhibit the QS and biofilm processes. One of them is a group of polyphenols, including flavonoids (naringenin, eriodyctiol, taxifolin, naringin, kaempferol, and quercetin), tannins, and limonoids (isolimonic acid, de-acetylnomilinic acid glucoside, and ichangin) [13]. These three compounds have a significant advantage, specifically to inhibit the autoinducer molecule in *P. aeruginosa* bacteria, known as AHL and HSL [14]. These three compounds were also able to inhibit various groups of genes encoding AHL formation, inhibit AHL signaling pathways through inactivation of this molecule when distributed in the bacterial cytoplasm, and reduce AHL concentrations by up to 40% through AHL-antagonist-like activity [15].

These three compounds are also found in grapefruit (*Citrus maxima*), also called pomelo in South-East Asia. Compared to other types of plants, such as lemon (*Citrus limon*), pomelo, especially in the peel, has a very high polyphenol content of 4.96% compared to 0.69% in lemon peel. In addition, 23% of the polyphenol content in pomelo is tannin. Moreover, as a group of citrus, pomelo has the highest limonoid content compared to other fruits [16]. This plant is spread throughout Indonesia, with the province of Bali contributing about 69.39% of the total national annual production [17]. With a high production rate, waste products such as fruit peels can be found easily. Therefore, based on the background described previously, the authors aimed to further investigate and evaluate the anti-QS and antibiofilm activities of pomelo peel on Multidrug-Resistant *P. aeruginosa*.

## 2. Method

**Fig. 1:**
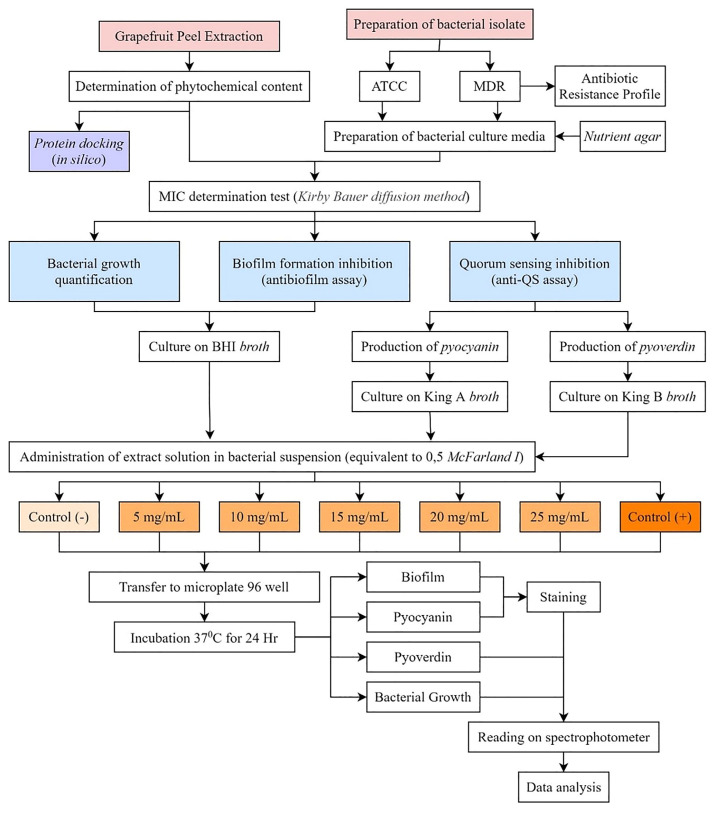
Research flow.

*Citrus maxima* (CM) peel was collected from a local farm in Denpasar, Bali, Indonesia. This study uses a clinical strain of *Pseudomonas aeruginosa* with high biofilm production and multidrug resistance (MDR) profile listed in supplementary data and ATCC as samples of biofilm formation. The extract of pomelo peel is prepared and performed with some assay methods related to MDR *P. aeruginosa*, including phytochemical analysis, bacterial growth with the Kirby-Bauer test, biofilm formation with the biofilm assay, and quorum-sensing activity with pyocyanin and pyoverdine assay. The computation methods were then performed to investigate the specific compounds and explore the molecular interaction of the extract with molecules related to the QS *P. aeruginosa* mechanism. The overall research flow has been demonstrated in Fig. 1.

### 2.1. *Citrus maxima* extract preparation

The peel of fresh grapefruit (*Citrus maxima*) was separated from the outer green (exocarp) and white (mesocarp) skin, followed by washing with clean water. Then, the material is cut into small pieces 5 cm in diameter, washed again, and steam blanched at 85 °C for 15 minutes. Then, the results that have been drained will be mashed using a dry blender and filtered twice using 80 mesh filter paper to get the peel slices of the grapefruit (*Citrus maxima*). Storage is carried out in closed and dry containers.

The peel extract of pomelo was prepared using the multistage maceration method (maceration at least twice) using ethanol 95% as a solvent with a ratio of 1:10 (w/v) measured using an analytical balance. The maceration was carried out twice (remaceration) with a total duration of 48 hours and concentrated by evaporation of ethanol through a rotary evaporator at a temperature of 40 °C, 200 mBar at a speed of 60 rpm for 120 minutes, and then put into the oven to remove the remaining solvent until a final weight was achieved.

### 2.2. Phytochemical analysis

We determined the phytochemical content of the peel extract by using phytochemical analysis. The purity of the bioactive fractions was determined using a thin-layer chromatography (TLC) plate. TLC was performed in a hexane: ethyl acetate: methanol (3:4:3) system.

### 2.3. Preparation of bacterial culture

The agar medium for bacterial culture and growth was prepared by pouring the sterile Mueller Hinton (MH) medium at a temperature of 50 °C into a Petri dish. Furthermore, the suspension of the test bacteria, namely MDR and ATCC *P. aeruginosa* was made, which was equivalent to a solution of 0.5 Mc Farland I (1.5 × 108 CFU/mL), then aseptically applied to the media. Finally, the medium was incubated for 24 hours at 37 °C.

### 2.4. Minimal inhibition concentration

The minimal inhibition concentration test of the extract against the test bacteria was carried out based on the Kirby Bauer diffusion (paper disk diffusion) method. The active extract was diluted with water as a solvent and made into a series of concentrations, namely 100, 200, 300, 400, and 500 mg/mL, with a total solution of 1 mL. Furthermore, each concentration of 0.02 mL of extract was dripped into a paper disk that had been placed in a sterile Petri dish, with repetitions at each concentration carried out 3 times (triplet). Positive control was made with the addition of the antibiotic ceftriaxone (30 mg). The negative control was only filled with water as a solvent in the form of distilled water. Then, the paper disk was placed on the culture medium and incubated for 24 hours in the incubator. The determination of the minimum inhibitory concentration is the minimum extract concentration that produces the largest diameter inhibition zone.

Bacterial growth was also measured by spectrophotometry at a wavelength of 600 nm to view the growth graph.

### 2.5. Biofilm assay

The bacterial suspension of MDR and ATCC *P. aeruginosa* containing 5, 10, 15, 20, 25 mg/mL and the control group in the form of medium solution and bacterial suspension (+) were put into sterile 96-well plates (flat bottom) and incubated for 48 hours at 37 °C. Then, the 96-well plates were rinsed with water and added 125 μL of 0.1% crystal violet solution to each well, and incubated plates at room temperature for 10–15 minutes. Then, the microplate was rinsed with water 3–4 times and dried for 4 hours. Finally, 125 μL of 30% acetic acid was added to solubilize the stain and the OD was measured at a wavelength of 550 nm. All tests were carried out using repetitions at each concentration, carried out 3 times (triplet). The percentage of biofilm inhibition can be calculated by the formula below.


$$\%\mathrm{Biofilm}\;\mathrm{inhibition}=\frac{{\text{OD}}_{\text{control}}-{\text{OD}}_{\text{experiment}}}{{\text{OD}}_{\text{control}}}\times 100\%$$

### 2.6. Pyocyanin and pyoverdine assay

Examination of the antiquorum-sensing effect used parameters such as extract inhibition on the production of quorum-sensing-dependent virulent factors (pyocyanin and pyoverdine pigment).

First, one colony of MDR *P. aeruginosa* bacteria was inoculated into a microplate containing King A broth for pyocyanin and King B broth for pyoverdine with a series of extract concentrations, namely 5, 10, 15, 20, and 25 mg/mL, and the control group culture medium (−) and bacterial suspension (+) as much as 1 mL of the final solution. Subsequently, each microplate was incubated at 37 °C for 24 hours, followed by centrifugation at 3542*g* for 10 minutes. After that, the cell-free supernatant was taken and dissolved in 0.5 mL of chloroform homogeneously, and 0.2 mL of 0.2 M HCl was added and centrifuged at 6797 g for 10 minutes until it turned red. For the pyoverdine assay, cell-free supernatant was taken without the need for staining. A total of 0.1 mL of the solution was measured spectrophotometrically (OD 520 nm, pyocyanin; OD 400, pyoverdine). All tests were carried out using repetitions at each concentration, carried out 3 times (triplet). Percent (%) inhibition was measured by the same formula on biofilms.

### 2.7. Molecular docking

#### 2.7.1. Protein and ligand preparation

The *P. aeruginosa* QS receptors, LasR (PDB: 3ix3) and LasI (PDB: 1ro5) were retrieved from the Protein Data Bank (PDB) (https://www.rcsb.org/) with.pdb file format. Ten active compounds as ligands in *Citrus maxima* were retrieved from the PubChem chemical database (https://pubchem.ncbi.nlm.nih.gov/) with.sdf file format. The files were then converted to mol2 using BIOVIA Discovery Studio for readability by iGEMDOCK.

#### 2.7.2. Docking

Docking was performed using iGEMDOCK version 2.1. iGEMDOCK offers dockings site from the specific binding site as docking with RMSD exceeding 2.0 are automatically excluded. The binding site used for LasR was OHN and none for LasI. The setting uses accurate docking, which offers the most accurate result with 800 populations in 80 generations. The result is in the form of the docking score and the interaction profile which are then visualized by using BIOVIA Discovery Studio for 2D interaction profile results and CHIMERA version 1.31 for 3D visualization.

### 2.8. Data analysis

Data that has been collected through observations will be processed by describing the results of the data characteristics in the form of tables and figures. The normality test used in this study is the Shapiro-Wilk test and the homogeneity of variance test uses the Levene test with SPSS software. The mean difference test was carried out using the One Way Anova test and continued with the Post Hoc Test to find out which groups were different. However, if the data is not normally distributed and not homogeneous, then a non-parametric test can be carried out, which is the Kruskal-Wallis Test. The Mann-Whitney test can be performed as a Post-Hoc test if the Kruskal-Wallis test shows p < 0.05.

## 3. Results

### 3.1. Phytochemical contents of *Citrus maxima* peel extract

According to our findings, the extract contained as much as 2104.45 mg of tannin, 1771.39 mg of flavonoid, and 2039.92 mg of phenol per 100 g extract, respectively.

### 3.2. Minimum inhibition concentration (MIC)

Based on the results of this study, no inhibition zone was found at an extract concentration of <500 mg/mL and positive control using ceftriaxone could be due to bacterial resistance in both ATCC and MDR strains. However, an inhibition zone was found at an extract concentration of 500 mg/mL with a minimum diameter of 6.4 mm and a maximum of 7.1 mm in MDR; 7.0 mm, and a maximum of 7.2 mm in the ATCC strain (see Table 1).

### 3.3. Maximum bacterial growth

**Fig. 2:**
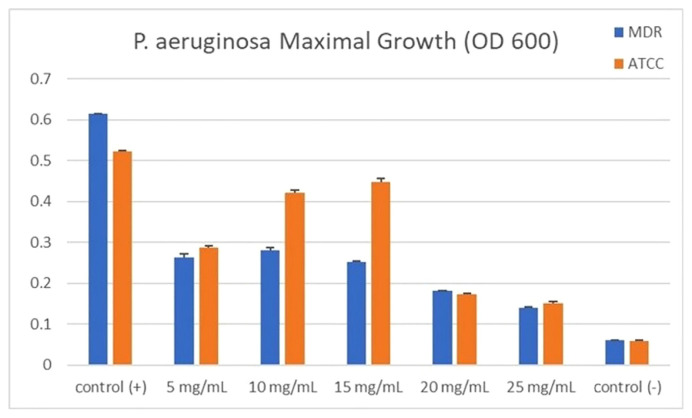
Maximum bacterial growth of *P. aeruginosa* (OD 600 nm).

Based on the results of this study, the extract concentration of 20–25 mg/mL significantly inhibited the bacterial growth of both MDR and ATCC strains (p 0.001), while the concentration of 5–15 mg/mL did not significantly inhibit the bacterial growth after 24 hours. In addition, the mean absorbance (OD 600 nm) of bacterial growth in the concentration series of 5–15 mg/mL versus 20–25 mg/mL was significantly different (p 0.001). This indicates that the best assessment of biofilm and quorum-sensing levels is at an extract concentration of between 5 and 15 mg/mL because it does not interfere with bacterial growth and therefore does not cause biased results as described in Table 2 and Fig. 2.

### 3.4. Biofilm biomass production

**Fig. 3:**
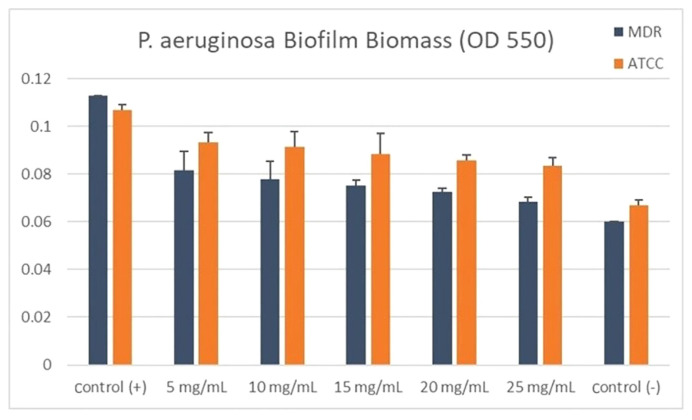
Maximum biomass production (OD 550 nm).

Based on the results of this study, we found that the biofilm production in both strains decreased significantly (p < 0.001) after 24 hours. The highest percentage of biofilm inhibition was in the group with a concentration of 25 mg/mL. However, this can be caused by the inhibition factor of bacterial growth; alternatively, using a concentration of 15 mg/mL. The One-Way ANOVA test showed that there was a significant difference among the groups tested (p<0.001) in both MDR and ATCC strains (Table 3 and Fig. 3).

As described in supplementary data, post hoc analysis also showed a significant difference between concentration and control groups. The unpaired *t*-test also showed that there were no significant differences in the absorbance values of the MDR group with ATCC, which indicated that the concentration of the extract effectively inhibited both types of strains as described in Table 4.

### 3.5. Biofilm inhibition (%)

Our study showed the highest proportion of inhibition (71% ± 4% in MDR (p < 0.001) and 47% ± 26% in ATCC (p < 0.001)) which was higher compared to previous studies using natural compounds, especially in MDR strains as described in Table 5.

### 3.6. Quorum sensing-related protein production (pyocyanin and pyoverdine)

Based on the results of this study, the absorbance value of both pyocyanin and pyoverdine in the concentration groups at a concentration of 5 mg/mL decreased significantly compared to the control group (p < 0.001). This shows that the administration of 5 mg/mL extract can inhibit the production of pyocyanin and pyoverdine MDR P. aeruginosa. We did not measure at a concentration > 5mg/mL because no bacterial growth in the culture broth was found at higher concentrations. The One-Way ANOVA test, as described in Table 6, revealed a significant difference between groups (p < 0.001).Based on the LSD post hoc test, there was a significant mean difference between each group in the pyocyanin (Table 7.) and pyoverdine (Table 8.) groups. Giving extract with a concentration of 5 mg/mL can inhibit pyocyanin and pyoverdine with an average percentage of inhibition of 44.9% ± 22.2% and 53.9% ± 22.9% in the MDR strain.

### 3.7. Molecular docking

Based on the results of the literature study, there were 10 compounds in CM that had antibiofilm or anti-quorum sensing effects, as described in the supplementary data (Table 14). A total of 9 and 4 compounds are known to be able to bind spontaneously to the active site of the LasI and LasR receptor proteins, which are indicated by negative binding energies, respectively. Furthermore, only naringin in LasI and quercetin in LasR had similar binding energy values to the positive control, namely ceftriaxone, as described in Table 9 and Table 10 (see Table 11).

**Fig. 4:**
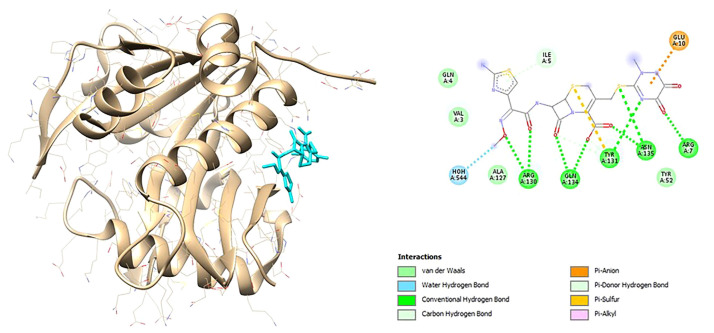
3D (left) and 2D (right) visualization of the ceftriaxone with LasI binding site.

**Fig. 5:**
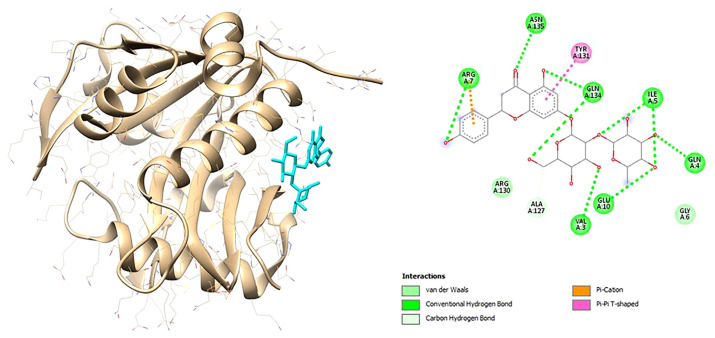
3D (left) and 2D (right) visualization of the naringin with LasI binding site.

**Fig. 6:**
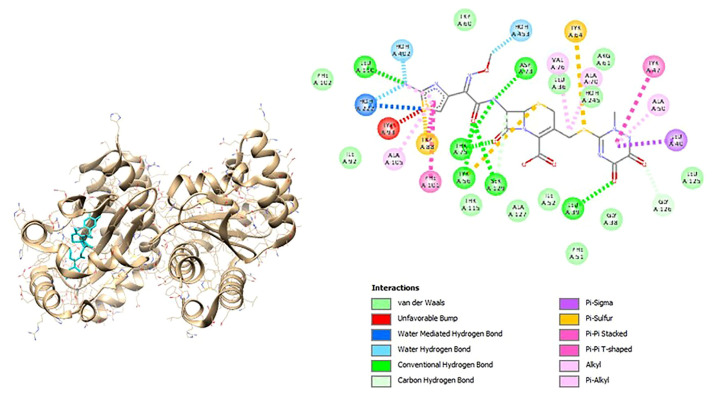
3D (left) and 2D (right) visualization of the ceftriaxone with LasR binding site.

**Fig. 7:**
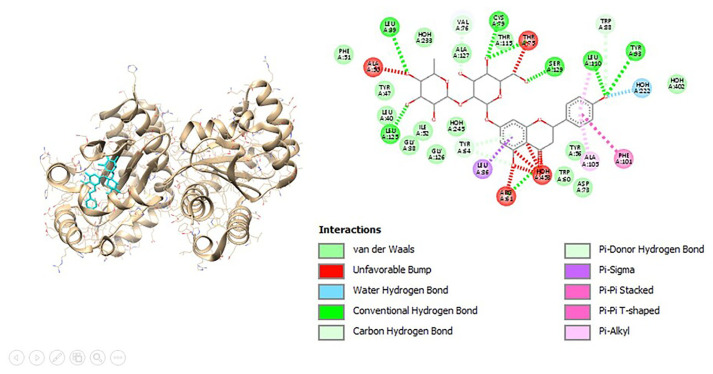
3D (left) and 2D (right) visualization of the naringin with LasR binding site.

The visualization results also show the interaction of organic components and chemical structures that are similar between these natural compounds and the control (ceftriaxone) and natural protein ligands as shown in Fig. 4 and Fig. 5 for Lasl and Fig. 6 and Fig. 7 for LasR.

## 4. Discussion

### 4.1. Minimum inhibitory concentration

Based on our study, we did not find any inhibition zones on ATCC and MDR *Pseudomonas aeruginosa* in the *Citrus maxima* peel extract group (<500 mg/mL) or in a positive control group with ceftriaxone due to bacterial resistance. However, on the administration of *Citrus maxima* peel extract with a concentration of 500 mg/mL, an inhibition zone was found with a minimum value of 6.4 mm on MDR and a maximum of 7.2 mm on ATCC. These results prove that *Citrus maxima* peel extract with a concentration of 500 mg succeeded in inhibiting ATCC and MDR *P. aeruginosa*, which were even resistant to the ceftriaxone antibiotic. A previous study by Abirami et al. (2013) also obtained similar results; namely, there was an 8–9 mm inhibition zone of *P. aeruginosa* bacteria with 50 mg/mL of *Citrus maxima* peel extract [18]. Another study by Ayad et al. (2017) also found an inhibition zone of *P. aeruginosa* of 10 mm in the group given *Citrus maxima* peel extract with concentrations of 75% and 95% [19].

The inhibition of *P. aeruginosa* by *Citrus maxima* peel extract was caused by its high flavonoid content. Flavonoids have antimicrobial effects through several mechanisms, such as inhibition of nucleic acid synthesis, inhibition of cytoplasmic membrane function by influencing biofilm formation, and interactions with various enzymes [20]. *Citrus maxima* peel contains various types of flavonoids, such as apigenin, kaempferol, and quercetin. Apigenin, which interacts with bacterial membranes, can cause bacterial cell lysis, and it was found that apigenin can inhibit *P. aeruginosa* bacteria with an inhibition zone of 17.5 mm [21,22]. Based on the study by Huang et al. (2015), kaempferol also has antibacterial effects, one of which is by inhibiting the activity of dihydropyridines in *P. aeruginosa*. [23] Quercetin compounds can work synergistically with antibiotics such as levofloxacin, gentamicin, and ceftriaxone in inhibiting biofilm formation (≥80%) and reducing infection rates significantly in *P. aeruginosa*. [24].

### 4.2. Inhibition of biofilm in *P. aeruginosa*

Our study showed that *Citrus maxima* extract has a statistically significant inhibition of biofilm in both the MDR and ATCC *P. aeruginosa*. The results of the phytochemical analysis in this study showed that the extract contained tannins, flavonoids, and phenols, respectively, of 2104.45 mg, 1771.39 mg, and 2039.92 mg per 100 g of extract. In the previous study, *Citrus maxima* showed it contains flavonoids, mainly naringin and aglycone naringenin [25]. Naringin, a glycoside of naringenin, was also studied for its effect as an antibiofilm. Naringin demonstrated antibiofilm activity and enhances the efficacy of the antibiotics ciprofloxacin and tetracycline in combination. Naringin inhibits biofilm formation by 49.5% in *P. aeruginosa*, which is shown by decreasing crystal violet staining in the biofilm assay, the extracellular polymeric substance production, and the metabolic activity of biofilm. Naringin also inhibits the motility of *P. aeruginosa* by decreasing the swarming and swimming of bacteria [26]. One of the polyphenolic compounds, gallic acid, was also found in sufficient quantity in the ethanol extract of *Citrus maxima* peel extract [27]. Gallic acid was found to inhibit biofilm formation and motility in *P. aeruginosa* by decreasing the biofilm formation in crystal violet or safranin staining of biofilm assay. Gallic acid decreases the motility of *P. aeruginosa* in swarming and twitching in motility assay [28]. Gallic acid was also reported to promote a reduction in biofilm activity by more than 70% [29].

Apigenin-8-C-glucoside (vitexin) was also found in citrus [30]. In a similar study, vitexin could inhibit biofilm formation in *P. aeruginosa* by decreasing biofilm formation in the crystal violet staining of the biofilm assay [31]. In another study, citrus was also reported to contain kaempferol [32]. Kaempferol could decrease the expression of genes involved in biofilm formation in *P. aeruginosa*. The type IV and flagellin type B (fliC) genes’ downregulation in response to kaempferol observed in RNA-seq was paralleled by the decreased expression of genes which are involved in biofilm formation in *P. aeruginosa* [33]. Rutin, which is also found in *Citrus maxima*, showed a concentration-dependent reduction of biofilm formation. Rutin decreases the total protein content and metabolic activity of biofilm-forming using the MTT assay to assess biofilm formation. However, it did not significantly decrease the biomass, while it reduced the secretion of extracellular polymeric substances. Therefore, it probably acts by interfering with adhesion and with all the other functions of extracellular polymeric substances [34,35].

Similar results were also obtained in a study conducted by Hnamte et al. (2018) in which there was a significant reduction in biofilm formation in the group given the extract *Plectranthus tenuiflorus*, which contains flavonoids, compared with a thick biofilm and solid-generated in the group controls, who were not given the extract. Biofilm formation in *P. aeruginosa* PAO1 was found to be reduced by 44% on treatment with *P. tenuiflorus* extract at a sub-MIC dose (500 μg/mL) [36]. In another study, the effect of antibiofilm was also evaluated with the addition of leaf extract from *Juglans regia* containing active compounds of phenolic acids and flavonoids on *P. aeruginosa*. The study also gave similar results, i.e., the inhibition of biofilm formation due to being given the extract. The comparison of the biofilm formation rates between the groups containing the culture and extract was *J. regia* 60% less than the group control without the extract [37]. In our study, it was found that *Citrus maxima* extract was able to inhibit biofilm formation by 70% on MDR *P. aeruginosa*.

These results may occur due to inhibition of gene regulators, such as *LasR* and *LasI*, by active compounds of *Citrus maxima*. Regulation of biofilm formation in *P. aeruginosa* can be influenced by various factors, and most of these mechanisms involve the quorum sensing system that acts as a regulatory component of GacS/GacA and RetS/LadS, exopolysaccharides, and c-di-GMP [38]. Quorum sensing is a communication process between cells in bacteria to regulate the expression of certain genes in response to changes in the density of the cell population. *P. aeruginosa* has three main quorum sensing systems, i.e., LasI-LasR, RhlI-RhlR, and PQS-MVFR, and all of them contribute to the formation of mature and differentiated biofilms that can maintain population density according to conditions [9].

### 4.3. Inhibition of pyoverdine and pyocyanin in *P. aeruginosa*

In our study, it was found that *Citrus maxima* peel extract with a concentration of 5 mg/mL could significantly inhibit the virulence factors pyocyanin and pyoverdine of MDR *Pseudomonas aeruginosa*. Based on a previous study, *Citrus maxima* peel flavonoid extract 0.9 and 1.8 mg REs/mL could inhibit violacein production by up to 73.69% and 80.62% in *C. violaceum* 026 without affecting bacterial growth, which indicates that the inhibition of violaceum is caused by an antiquorum-sensing mechanism. *Citrus maxima* peel extract is also able to reduce the production of auto inducer (AI)-2 in *V. anguillarum* by up to 45.99% by decreasing the mRNA expression of the VanS gene that regulates AI-2 production. Al-2 is a quorum-sensing signal used for communication and a regulator of virulence factors of various bacterial pathogens up to 45.99% by decreasing the mRNA expression of the VanS gene that regulates AI-2 production. Al-2 is a quorum-sensing signal used for communication and a general regulator of virulence factors of various bacterial pathogens [39].

*Citrus maxima* contains various flavonoid compounds, some of which are quercetin, naringenin, and naringin. The study by Paczkowski et al. (2017) found that flavonoids that have a dihydroxyl moiety such as quercetin can reduce virulence factors in the quorum sensing mechanism, so that they can inhibit quorum sensing through interaction with LasR [40]. Quercetin (16 μg/mL) can reduce pyocyanin production by 58% and significantly reduce the expression of the *lasI, lasR, rhlI*, and *rhlR* genes required for quorum sensing [41]. Based on the study of Vandeputte et al. (2011), the production of quorum sensing virulence factors, namely pyocyanin and elastase, also decreased in the group given naringenin (4 mM). Naringenin was found to inhibit the production of AHL molecules and the regulation of the *lasl* and *rhll* genes that express *lasB* (encoding elastase), *rhlA* (involved in the formation of rhamnolipids), and the phz operon that is involved in pyocyanin production. This inhibition is caused by the presence of a hydroxyl component in naringenin, which is different from other flavonoid compounds. In this study, no significant antiquorum sensing effect was found by naringin, but in another in silico study, the binding affinity of naringin with the LasR receptor protein is −16.53 kcal/mol [39,42].

In our study, administration of *Citrus maxima* bark extract at a concentration of 5 mg/mL inhibited pyocyanin and pyoverdine by 45% and 54%, respectively. This result is probably due to the inhibition of several components of the quorum-sensing mechanism, such as the gene regulators LasI and LasR. Gene I codes for autoinducers or AHL such as OdDHL and BHL, while gene R encodes LasR and RhlR receptors. By inhibiting the formation of autoinducer and receptor, the LasR-OdDHL and RhlR-BHLL complexes are not formed. These complexes are required for the activation of gene expression for specific factors such as virulence, sporulation, pigmentation, and biofilm formation. Pyocyanin and pyoverdine are virulence factors of the quorum sensing mechanism in *Pseudomonas aeruginosa* [9].

## 5. Conclusion

Our in vitro and in silico studies suggest a significant antiquorum-sensing and antibiofilm effect of pomelo peel (*Citrus maxima*) extract on Multidrug-Resistance (MDR) *Pseudomonas aeruginosa*. Inhibition of biofilm formation by pomelo peel extract was found to be significant. Moreover, the antiquorum-sensing activity in MDR through inhibition of pyocyanin and pyoverdine production was found to be significant. In addition, our molecular docking suggests no significant difference in the binding energy of polyphenolic compounds found in pomelo peel with QS-related ligans (LasR and LasI) compared to native ligands and inhibitors, which indicates that QS activity can be influenced by polyphenolic compounds of pomelo peel. This finding determines pomelo peel (*Citrus maxima*) extract as a potential pharmacological agent for MDR *Pseudomonas aeruginosa*-related infections. Along with that, the extract can be combined to improve antibiotic potency as bacteriostatic and/or bactericidal agents. Further research is needed to determine the synergistic effect between the combination of antibiotics and *Citrus maxima* peel extracts as an effective and efficient antimicrobial agent.

## Figures and Tables

**Table 1:** Phytochemical contents of *C. maxima* peel extract.

No	Compounds	Content (mg/100g extract)
1	Tannin	2104.45
2	Flavonoid	1771.39
3	Phenol	2039.92

**Table 2:** Maximum bacterial growth of *P. aeruginosa* (OD 600 nm).

No	Group	MDR	ATCC
1	Control (+)	0.62 ± 0.01	0.52 ± 0.01
2	5 mg/mL	0.26 ± 0.26	0.29 ± 0.01
3	10 mg/mL	0.28 ± 0.38	0.42 ± 0.04
4	15 mg/mL	0.25 ± 0.14	0.45 ± 0.20
5	20 mg/mL	0.18 ± 0.09	0.17 ± 0.02
6	25 mg/mL	0.14 ± 0.02	0.15 ± 0.02
7	Control (−)	0.06 ± 0.17	0.06 ± 0.06

**Table 3:** One way ANOVA of biofilm biomass production (OD 550 nm).

No	Group	MDR		ATCC	
	
		Mean ± Std	p-value	Mean ± Std	p-value
1	Control (+)	0,060 ± 0,000	p < 0.001	0.067 ± 0.002	p < 0.001
2	5 mg/mL	0,0817 ± 0,007		0.093 ± 0.004	
3	10 mg/mL	0,078 ± 0,006		0.091 ± 0.006	
4	15 mg/mL	0,075 ± 0,002		0.088 ± 0.009	
5	20 mg/mL	0,073 ± 0,001		0.073 ± 0,001	
6	25 mg/mL	0,068 ± 0.002		0.084 ± 0.003	
7	Control (−)	0,113 ± 0,000		0.107 ± 0.002	

**Table 4:** Unpaired *t*-test of Biofilm Biomass Production between MDR and ATCC strain.

No	Group	MDR	ATCC	Mean difference (CI 95%)	p-value
1	Control (−)	0.060 ± 0.000	0.067 ± 0.002	0.007 (0.003–0.010)	0.004
2	5 mg/mL	0.082 ± 0.008	0.093 ± 0.004	0.012 (0.002–0.026)	0.088
3	10 mg/mL	0.078 ± 0.007	0.091 ± 0.006	0.013 (0.003–0.029)	0.080
4	15 mg/mL	0.075 ± 0.002	0.088 ± 0.008	0.013 (0.001–0.027)	0.065
5	20 mg/mL	0.072 ± 0.001	0.085 ± 0.002	0.013 (0.008–0.018)	0.002
6	25 mg/mL	0.068 ± 0.002	0.083 ± 0.003	0.015 (0.009–0.021)	0.002
7	Control (+)	0.113 ± 0.000	0.107 ± 0.002	0.006 (0.003–0.009)	0.007

**Table 5:** Percentage of biofilm inhibition.

No	Group	Mean ± Std (%)	

		MDR	ATCC
1	05 mg/mL	59.12 ± 15.13	33.84 ± 9.64
2	10 mg/mL	66.03 ± 14.24	38.65 ± 18.08
3	15 mg/mL	71.06 ± 4.35	47.14 ± 25.64
4	20 mg/mL	76.10 ± 2.88	53.13 ± 7.70
5	25 mg/mL	84.28 ± 3.93	58.56 ± 11.84

**Table 6:** One way ANOVA of pyocyanin (OD 560 nm) and pyoverdine production (OD 400 nm).

No	Group	Pyocyanin		Pyocyanin	
	
		Mean ± Std	p-value	Mean ± Std	p-value
1	Control (−)	0.335 ± 0.074	p < 0.001	0.074 ± 0.003	p < 0.001
2	5 mg/mL	0.573 ± 0.030		0.302 ± 0.083	
3	Control (+)	0.784 ± 0.030		0.495 ± 0.057	

**Table 7:** Post hoc LSD of pyocyanin production.

	Mean difference	CI 95%		p-value

		Minimum	Maximum	
c (−) vs 5 mg/mL	− 0,2286	− 0,3452	−0,112	p = 0,003
5 mg/mL vs c (+)	−0,1926	− 0,3092	− 0,76	p = 0,007
c (−) vs c (+)	−0,4213	− 0,3047	− 0,5379	p < 0,001

**Table 8:** Post hoc LSD of pyoverdine production.

	Mean difference	CI 95%		p-value

		Minimum	Maximum	
c (−) vs 5 mg/mL	− 0,2376	− 0,3525	− 0,1227	p = 0,002
5 mg/mL vs c (+)	− 0,2113	− 0,3262	− 0,0964	p = 0,004
c (−) vs c (+)	− 0,449	− 0,334	− 0,5639	p < 0,001

**Table 9:** Bonding energy of natural compound found in CM with LasR.

No.	Compound	Energy	VDW	H Bond
1.	Apigenin	−117.846	−98.3592	−19.4872
2.	Vitexin	−130.563	−92.9344	−37.6287
3.	Diosmetin	−117.548	−95.2856	−22.2625
4.	Eriodictyol	−122.568	−98.7682	−23.7996
5.	Kaempferol	−120.385	−99.0249	−21.3597
6.	Naringenin	−114.911	−96.2438	−18.667
**7**.	**Naringin**	**−162.599**	**−130.035**	**−32.5638**
8.	Quercetin	−121.004	−83.8868	−37.1177
9.	Rutin	−149.605	−95.2019	−54.4031
**10**.	**Ceftriaxone**	**−159.133**	**−124.199**	**−34.9344**

The bold values indicate similar energy binding between the C. maxima compound with the current treatment.

**Table 10:** Bonding energy of natural compound found in CM with LasI.

No.	Compound	Energy	VDW	H Bond
1.	Naringin	−134.414	−97.428	−36.9856
**2**.	**Quercetin**	**−140.492**	**−97.1138**	**−43.3783**
3.	Rutin	−148.733	−112.887	−35.8459
4.	Vitexin	−113.09	−56.7046	−56.3859
**5**.	**Ceftriaxone**	**−140.342**	**−87.8707**	**−52.2228**

The bold values indicate similar energy binding between the C. maxima compound with the current treatment.

**Table 11:** Naringin, gallic acid, and vitexin inhibiting biofilm formation and motility in *P. aeruginosa*. [26,28,30].

Compounds	Biofilm Assay				Motility Assay		
	
	Crystal Staining	Violet/Safranin	Metabolic Activity of Biofilm	Extracellular Polymeric Substance Production	Swarming	Swimming	Twitching
Naringin	↓ 49.5%		↓ 49.5%	↓ 40%	↓ 42%	↓ 14%	
Gallic Acid	↓ 2–2.5-fold				↓ 20–50%		↓ 0–15%
Vitexin	↓ 56%			↓ 40%	↓ 100μ		

## Appendix Supplementary data

**Table 12:** Post hoc LSD of biofilm inhibition in MDR *P. aeruginosa*.

	Mean difference	CI 95%		p value

		Minimum	Maximum	
c (−) vs 5 mg/mL	− 0,0217	− 0,0293	− 0,0140	p < 0,001
c (−) vs 10 mg/mL	− 0,0180	− 0,0256	− 0,0104	p < 0,001
c (−) vs 15 mg/mL	− 0,0153	− 0,0230	− 0,0077	p = 0,001
c (−) vs 20 mg/mL	− 0,0127	− 0,0203	− 0,0050	p = 0,003
c (−) vs 25 mg/mL	− 0,0083	− 0,0606	− 0,0454	p = 0,035
c (−) vs c (+)	− 0,0530	− 0,0606	− 0,0454	p < 0,001
5 mg/mL vs 10 mg/mL	0,0037	− 0,0040	0,0113	p = 0,321
5 mg/mL vs 15 mg/mL	0,0063	− 0,0013	0,0140	p = 0,097
5 mg/mL vs 20 mg/mL	0,0090	0,0014	0,0166	p = 0,024
5 mg/mL vs 25 mg/mL	0,0133	0,0057	0,0210	p = 0,002
5 mg/mL vs c (+)	− 0,0313	− 0,0390	− 0,0237	p < 0,001
10 mg/mL vs 15 mg/mL	0,0027	− 0,0050	0,0103	p = 0,467
10 mg/mL vs 20 mg/mL	0,0053	− 0,0023	0,0130	p = 0,157
10 mg/mL vs 25 mg/mL	0,0097	0,0020	0,0173	p = 0,017
10 mg/mL vs c (+)	− 0,0350	− 0,0426	− 0,0274	p < 0,001
15 mg/mL vs 20 mg/mL	0,0027	− 0,0050	0,0103	p = 0,467
15 mg/mL vs 25 mg/mL	0,0070	− 0,0006	0,0146	p = 0,070
15 mg/mL vs c (+)	− 0,0377	− 0,0453	− 0,0300	p < 0,001
20 mg/mL vs 25 mg/mL	0,0043	− 0,0033	0,0120	p = 0,244
20 mg/mL vs c (+)	− 0,0403	− 0,0480	− 0,0327	p < 0,001
25 mg/mL vs c (+)	− 0,0447	− 0,0523	− 0,0370	p < 0,001

**Table 13:** Post hoc LSD of biofilm inhibition in ATCC *P. aeruginosa*.

	Mean difference	CI 95%		p-value

		Minimum	Maximum	
c (−) vs 5 mg/mL	− 0,0264	− 0,3462	− 0,018	p < 0,001
c (−) vs 10 mg/mL	− 0,0244	− 0,3262	− 0,016	p < 0,001
c (−) vs 15 mg/mL	− 0,0214	− 0,2962	− 0,013	p < 0,001
c (−) vs 20 mg/mL	− 0,0187	− 0,2695	− 0,0104	p < 0,001
c (−) vs 25 mg/mL	− 0,0167	− 0,2495	− 0,0084	p = 0,001
c (−) vs c (+)	− 0,04	− 0,0428	− 0,0317	p < 0,001
5 mg/mL vs 10 mg/mL	0,002	− 0,0062	0,0103	p = 0,613
5 mg/mL vs 15 mg/mL	0,005	− 0,0033	0,0133	p = 0,216
5 mg/mL vs 20 mg/mL	0,0077	− 0,0006	0,016	p = 0,067
5 mg/mL vs 25 mg/mL	0,0097	0,0014	0,018	p = 0,025
5 mg/mL vs c (+)	− 0,0137	− 0,022	− 0,0054	p = 0,003
10 mg/mL vs 15 mg/mL	0,003	− 0,0053	0,0113	p = 0,450
10 mg/mL vs 20 mg/mL	0,0057	− 0,0026	0,014	p = 0,164
10 mg/mL vs 25 mg/mL	0,0077	− 0,0006	0,016	p = 0,067
10 mg/mL vs c (+)	− 0,0157	− 0,2395	− 0,0074	p = 0,001
15 mg/mL vs 20 mg/mL	0,0027	− 0,0056	0,011	p = 0,501
15 mg/mL vs 25 mg/mL	0,0047	− 0,0036	0,013	p = 0,247
15 mg/mL vs c (+)	− 0,0187	− 0,027	− 0,0104	p < 0,001
20 mg/mL vs 25 mg/mL	0,002	− 0,0063	0,0103	p = 0,613
20 mg/mL vs c (+)	− 0,0214	− 0,0296	− 0,013	p < 0,001
25 mg/mL vs c (+)	− 0,024	− 0,0316	− 0,015	p < 0,001

**Table 14:** Antimicrobial activity of *Citrus maxima* compounds.

Compound	Compound class	Effects	Reference
Apigenin	Flavonoid	Antiquorum-sensing	(Dong et al., 2013)
Diosmetin	Flavonoid	Antiquorum-sensing	(Liu et al., 2015)
Eriodictyol	Flavonoid	Antiquorum-sensing	(Vandeputte et al., 2011)
Gallic acid	Tannin	Antibiofilm	(Borges et al., 2012; Plyuta et al., 2013)
Kaempferol	Flavonoid	Antibiofilm	(Ming et al., 2017)
Naringenin	Flavonoid	Antiquorum-sensing and Antibiofilm	(Dey et al., 2020; Vandeputte et al., 2011)
Naringin	Flavonoid	Antiquorum-sensing and Antibiofilm	(Dey et al., 2020; Vandeputte et al., 2011)
Quercetin	Flavonoid	Antiquorum-sensing	(Carrada López & Castañón Sánchez, 2018)
Rutin	Flavonoid	Antibiofilm	(Al-Shabib et al., 2017)
Vitexin	Flavonoid	Antiquorum-sensing and Antibiofilm	(Das et al., 2016)
